# Negative Affective States Are Not Detected in Rats Following an Intravenous Self‐Administration Regimen Leading to Incubation of Oxycodone Craving

**DOI:** 10.1111/adb.70196

**Published:** 2026-11-02

**Authors:** Amanda M. Wunsch, Kimberley A. Mount, Asia Guzman, Alex B. Kawa, Jonathan G. Westlake, Hayley M. Kuhn, Madelyn M. Beutler, Marina E. Wolf

**Affiliations:** ^1^ Department of Behavioral Neuroscience Oregon Health & Science University Portland Oregon USA

**Keywords:** incubation of craving, negative affect, oxycodone, rat, self‐administration, somatic withdrawal signs

## Abstract

In rats, cue‐induced opioid craving intensifies (incubates) during abstinence from opioid self‐administration and then remains high for a prolonged period. The prolonged plateau models persistent vulnerability to cue‐induced craving and relapse in humans recovering from opioid use disorder. However, a very significant contributor to relapse vulnerability in these individuals is the presence of negative affective states that can persist for months to years, far beyond physical dependence. The goal of this study was to determine, for oxycodone, if the incubation of craving model recapitulates this aspect of relapse vulnerability. We began by comparing rats trained to self‐administer oxycodone using a regimen leading to persistent elevation of cue‐induced craving (6 h/day × 10 days) and rats trained to self‐administer saline. We assessed somatic withdrawal signs in early abstinence and conducted behavioural tests modelling negative affect (open field, social preference, sucrose preference and elevated plus maze) in late abstinence. Some oxycodone rats exhibited somatic withdrawal signs on abstinence day (AD)1. However, on AD41–46, no group differences were found in behavioural tests modelling negative affect. To compare early and late abstinence periods, a second cohort of rats self‐administered saline or oxycodone and then received a sequence of tests (cue‐induced seeking test followed by negative affect tests) in early abstinence (AD1–7) and again in late abstinence (AD40–46). While some time‐dependent changes in affect were observed within each group, they did not indicate emergence of negative affect in oxycodone rats during incubation of craving. In conclusion, our results suggest that rats exhibiting incubation are not seeking the drug to relieve an aversive state that persists into protracted abstinence. An alternative explanation is that incubated seeking reflects increased incentive salience of the drug‐paired cue. Our finding of modest somatic withdrawal signs in early abstinence and no detectable negative affective states in late abstinence probably reflects lower drug intake during the intravenous regimen compared to non‐contingent escalating dose regimens typically used to study withdrawal signs. Thus, while the incubation model has utility for studying cue‐induced oxycodone craving, our standard incubation regimen is not suited to studying oxycodone seeking driven by the desire to relieve an aversive state.

## Graphical abstract

This study asked whether negative affective states are detected in rats after protracted abstinence from an oxycodone self‐administration regimen leading to incubation of craving. After more than 40 days of abstinence, when incubation is robust, we found no differences between saline and oxycodone rats in tests of negative affect. Our results suggest that rats exhibiting incubation are not seeking oxycodone to relieve an aversive state that persists into protracted abstinence.

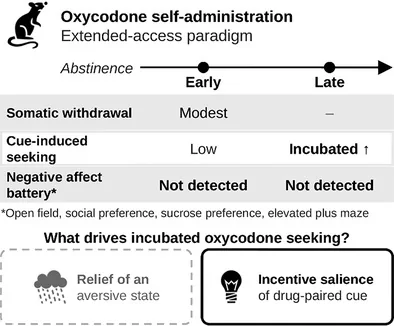

## 1 Introduction

In people with opioid use disorder (OUD), craving is strongly correlated with the likelihood of relapse [1], and this vulnerability to relapse persists during protracted abstinence [2]. Cue‐induced drug craving and relapse can be studied using the incubation of craving model. In rats, cue‐induced seeking for drugs of abuse (stimulants, nicotine, alcohol and opioids) progressively intensifies (incubates) during forced or voluntary abstinence from drug self‐administration (SA) and then remains high for a prolonged period [3]. In humans, incubation has been demonstrated during abstinence from cocaine, methamphetamine, nicotine and alcohol but remains controversial for opioids [3] (see Section 4). Nevertheless, persistence of craving during abstinence, captured by the plateau phase of the incubation model, applies across all drugs. The model's focus on cue‐induced craving is also translationally relevant. Abstinent opioid users exhibit robust motivational bias towards opioid‐associated stimuli [4], and such bias predicts heroin relapse [5] and is correlated with craving [6]. Thus, the incubation of craving model has translational relevance to certain aspects of OUD.

However, it is well established that negative affective states of opioid abstinence (including loss of motivation for natural rewards, anxiety, dysphoria and irritability) contribute very significantly to relapse vulnerability; importantly, they persist for months to years in recovering opioid users, far beyond somatic withdrawal (SWD) signs [7, 8]. In rodents, these states have been extensively studied after non‐contingent escalating dose opioid regimens leading to physical dependence [9, 10]. While physical dependence wanes in days to a week, negative affective states are more persistent and sometimes intensify from 1 to 4–6 weeks of withdrawal [9, 10]. Tests that often revealed progressively intensifying negative affective states included the open field test (OFT; center time), tests of social interaction such as the social preference test (SoPT), the elevated plus maze (EPM) and tests of motivation for natural rewards such as the sucrose preference test (SuPT) [11, 12, 13, 14, 15, 16, 17, 18, 19]. Far less is known about the persistence of negative affective states following opioid SA, as most such studies have focused on the early abstinence period (see Section 4).

The goal of this study was to determine if negative affective states can be detected after protracted abstinence in conjunction with incubation of opioid craving. If so, this would suggest the possibility that rats exhibiting incubation are seeking the drug in part to relieve an aversive state that persists into protracted abstinence. We focused on oxycodone, a widely prescribed opioid that is also widely abused [20] and that elicits incubation of craving in rats after forced or voluntary abstinence [3]. To assess negative affective states, we conducted the OFT, SoPT, SuPT and EPM (based on studies cited in the previous paragraph) after different periods of abstinence from saline or oxycodone SA. We used an extended‐access oxycodone regimen (0.1 mg/kg/infusion × 6 h/day × 10 days) that produced robust incubation in our previous study [21]. This oxycodone dose is commonly used in incubation studies across labs (recent examples: [22, 23, 24]).

## 2 Materials and Methods

### 2.1 Animals

We obtained rats on a Long‐Evans background (26 males and 28 females) from our in‐house breeding colony and used both sexes in each experiment. Our recent study [21] found similar oxycodone incubation in males and females using the same rat line and oxycodone regimen, in keeping with many but not all results in the literature (see Section 4.5). We housed two to three rats per cage until the time of jugular catheter surgery or single housing, at which time we individually housed rats. Rats were 7–15 weeks old at the start of the experiment. Rats had free access to food and water and were maintained on a 12‐h light/dark cycle. We excluded three rats for health‐related issues. All procedures were approved by the OHSU Institutional Animal Care and Use Committee and conform to guidelines outlined in the *Guide for the Use and Care of Animals* and *Policy on Humane Care and Use of Laboratory Animals*.

### 2.2 Jugular Catheter Surgery

Rats destined for oxycodone (or saline) SA received jugular catheter implants for drug delivery. We anaesthetized rats using isoflurane (5% induction, 2%–3% maintenance) and injected them with meloxicam (5 mg/kg, SC) for analgesia. We shaved surgical sites and prepared them with betadine and ethanol scrubs. Jugular catheters consisted of a mesh‐reinforced, 22‐gauge cannula with an attached 10‐cm silastic tube. We placed the exterior cannula on the back, threaded the tube subcutaneously around the shoulder blade and inserted the tube into the jugular vein. We applied topical antibiotics to the incisions and administered prophylactic cefazolin (30 mg/kg, IV). Rats received 3 days of post‐operative meloxicam and recovered from surgery for 5–8 days before the start of drug SA. We flushed catheters 5–7 days per week with heparinized saline (20 IU/mL, IV) until the cessation of drug SA.

### 2.3 Oxycodone SA

Rats self‐administered oxycodone (0.1 mg/kg/infusion; NIDA Drug Supply Program) or 0.9% saline for 6 h/day for 10 consecutive days under a fixed ratio 1 schedule as described previously [21]. Nose pokes in the active hole triggered the infusion pump and simultaneously activated a 4‐s cue light and time‐out period. Pokes in the inactive hole had no consequence. Following SA, rats remained in their home cage for the duration of forced abstinence except for periods of behaviour testing.

### 2.4 Cue‐Induced Seeking Tests

Rats returned to the SA chamber for 1 h. Nose pokes in the previously active hole resulted in presentation of the same cue light previously paired with drug but without drug infusion. Pokes in the inactive hole continued to result in no consequences. The number of pokes in the previously active hole is our measure of cue‐induced craving. To assess incubation, some rats received seeking tests on abstinence day (AD)1 and AD40, and an ‘incubation score’ was calculated (pokes in the previously active hole on AD40 − pokes on AD1), as in other studies (e.g., [22]).

### 2.5 Spontaneous Somatic Withdrawal (SWD) Symptoms

Assessments occurred in an acrylic cylinder (30 cm diameter × 50 cm tall) with bedding on the floor. Habituation to the cylinder occurred during two 10‐min sessions prior to SA. On AD1–7, we placed rats in the cylinder for 10 min while video‐recording behaviour. An experimenter blind to the experimental condition counted the frequency of digging, jumping, rearing, grooming and teeth chatters. We report results for each behaviour individually on AD1, AD2 and AD3. In an additional study (not shown), oxycodone and saline rats showed no difference in these behaviours on AD7. We also present an SWD score calculated as the sum of the total occurrences of each behaviour on AD1. We modified previous procedures [25, 26] to include only behaviours we could reliably detect from videos. We recorded rat weight prior to each session.

### 2.6 OFT

This test, which can be used to measure anxiety‐like behaviour [27], was performed in an arena with textured floors (100 × 100 × 45 cm). We placed rats in the center of the arena and allowed them to freely explore for 5 min. ANYmaze software tracked the center of the body and calculated the total distance travelled and the number of entries and time spent in the periphery (10 cm around the edge) and center (inner 80 cm^2^). The percentage of time spent in the center of the arena was used as a measure of anxiety‐like behaviour.

### 2.7 SoPT

This test, commonly used to study social motivation and reward [28], was carried out in an apparatus consisting of three chambers separated by walls with spaces that allow rats free access to all chambers. The outer two chambers are 50 × 60 cm, and the center chamber is 30 × 60 cm. Two wire cups are placed on opposite sides of the apparatus. During habituation, we placed the test rat in the center of the chamber and allowed it to freely explore the entire apparatus for 5 min. During the test phase, we placed a stranger conspecific (same sex as the test rat) under one of the wire cups (location of stranger conspecific was counterbalanced across test subjects). We then placed the test rat in the center of the chamber, and the rat freely explored the apparatus for 10 min. ANYmaze software tracked the center of the rat and calculated the number of entries into and time spent exploring the chamber with the stranger conspecific or the empty cup. We calculated the social preference score as the percentage of time the test rat spent exploring the stranger conspecific divided by total time spent exploring the stranger conspecific and empty cup.

### 2.8 SuPT

We used the SuPT as a measure of anhedonia or depressive‐like behaviour. We performed a 3‐day SuPT protocol as described previously [29] in the rat's home cage. To habituate the rats to the two‐bottle choice paradigm and overcome neophobia, rats had access to two bottles, one containing 1% sucrose solution and one that was empty, for 2 days. Rats did not consume normal water during this period. On Day 3, we removed both bottles from the cage, and rats underwent a 4‐h water deprivation period to increase motivation to consume liquid during the test period. During the 1‐h test, rats had access to one bottle containing 1% sucrose and a second bottle containing water. We gave rats fresh sucrose bottles and switched the location of the sucrose bottle daily. We measured sucrose and water consumption by weighing the bottles before and after the test and calculated sucrose preference as the amount of sucrose consumed over the total amount of sucrose and water consumed.

### 2.9 EPM

This test, widely used to measure anxiety‐like behaviour [27], was performed in an apparatus in the shape of a plus sign (+) raised 75 cm off the floor, with two arms encased by opaque walls (50 × 10 × 60 cm) and two open arms without walls (50 × 10 cm). We placed rats in the center of the apparatus and allowed them to freely explore for 5 min. ANYmaze software tracked the center of the body and calculated the number of entries and time spent in the center, closed and open arms. The percentage of time spent in the open arm relative to the total time spent in the open and closed arms was our measure of anxiety‐like behaviour.

### 2.10 Statistical Analyses

We used GraphPad Prism to plot data and perform statistical analyses. We tested for normal distribution using the Shapiro–Wilk test. Two‐way mixed ANOVAs were used to assess infusions taken during SA (drug and day as factors), nose pokes during both SA and cue‐induced seeking tests (hole and drug as factors), and time‐dependent changes in spontaneous SWD signs, body weight, active and inactive nose pokes during seeking tests, OF, SoPT, SuPT, and EPM data (AD and drug as factors). Significant interactions were followed up with uncorrected Fisher's LSD post hoc tests, and significant main effects were followed up using Sidak's multiple comparison's test. One‐way ANOVA followed by Tukey's multiple comparisons test or non‐parametric Kruskal–Wallis test followed by Dunn's multiple comparisons test were used to assess OF, SoPT, SuPT, and EPM in single‐housed naïve rats and rats that underwent oxycodone or saline SA. Unpaired *t*‐tests or non‐parametric Mann–Whitney *U* tests were used to assess average infusions taken during SA or OF, SuPT, SoPT, and EPM data in pair‐ and single‐housed rats. Rats of both sexes were used in all experiments, and we observed no obvious differences between males and females for any measure. Because these studies were not sufficiently powered to detect sex differences, data from both sexes are combined. All data are presented as mean ± SEM.

## 3 Results

### 3.1 Spontaneous SWD Symptoms and Behaviours Indicative of Negative Affect During Abstinence From Oxycodone or Saline SA

The major goal of the first experiment was to determine if our oxycodone SA regimen elicits negative affective states after protracted abstinence that might contribute to incubated oxycodone seeking; we also assessed SWD symptoms in early abstinence (timeline in Figure 1A). Key ANOVA results are reported here. For full ANOVA results, see Table 1 (for Figure 1 data) or Table S1 (for Figure S1 data); for post hoc test results, see legends to Figures 1 and S1.

**FIGURE 1:**
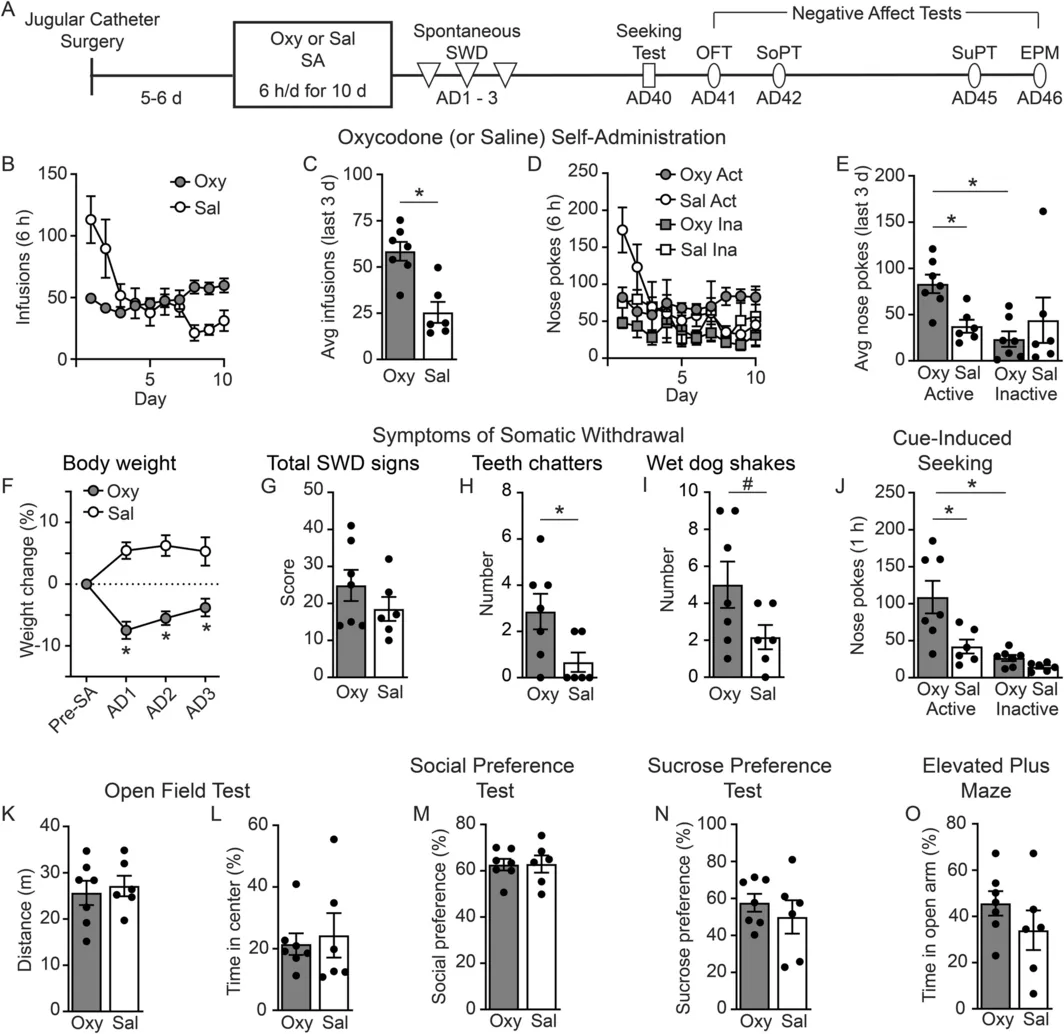
Oxycodone self‐administration (SA), symptoms of somatic withdrawal following cessation of oxycodone SA, and cue‐induced oxycodone craving and negative affective states after 40 days of forced abstinence. (A) Rats received jugular catheters and recovered for 5–6 days. Rats self‐administered oxycodone (Oxy; 0.1 mg/kg/infusion; *n* = 7) or saline (Sal; *n* = 6) for 6 h/day for 10 days. We assessed symptoms of spontaneous somatic withdrawal (SWD) on abstinence day (AD) 1–3 following SA. Rats then underwent a cue‐induced seeking test (1 h) on AD40, open field test (OFT) on AD41, social preference test (SoPT) on AD42, sucrose habituation on AD43–44, sucrose preference test (SuPT) on AD45 and elevated plus maze (EPM) on AD46. (B) The number of infusions of Oxy or Sal self‐administered in each 6‐h session across the 10 days of SA. During early days of training, several saline rats took a higher than typical number of infusions, but there was no reason for excluding these animals. (C) During the last 3 days of SA, Oxy rats took a significantly higher number of infusions than Sal rats (unpaired *t*‐test, *p* = 0.0012). (D) Nose pokes in the active (Act) and inactive (Ina) hole in rats that self‐administered Oxy or Sal. (E) During the last 3 days of SA, Oxy rats poked more in the active hole than the inactive hole (post hoc uncorrected Fisher's LSD, *p* = 0.0094), an effect not observed in Sal rats. Oxy rats poked more in the active hole than Sal rats (post hoc uncorrected Fisher's LSD, *p* = 0.0271). (F) Oxy rats exhibited opposite changes in body weight on AD1–3 compared to Sal rats (post hoc Sidak's multiple comparisons, Sal vs. Oxy on AD1–3, all *p* < 0.01 as indicated by asterisks), with the weight of Oxy rats significantly decreased on AD1–2 compared to their pre‐SA weight (post hoc Sidak's multiple comparisons, Oxy: pre‐SA vs. AD1 and AD2, *p* < 0.02). (G) The somatic withdrawal score (sum of occurrences of grooming bouts, wet dog shakes, teeth chatters, jumps and digging bouts in 10 min) did not differ between Oxy and Sal rats on AD1. (H) Oxy rats teeth‐chattered significantly more than Sal rats on AD1 (Mann–Whitney test, *p* = 0.0449). (I) Oxy rats trended towards an increase in the number of wet dog shakes compared to Sal rats on AD1 (unpaired *t*‐test, *p* = 0.0836). (J) Oxy rats poked in the previously active hole significantly more than Sal rats (post hoc Sidak's multiple comparisons, *p* = 0.0029) and in the inactive hole (post hoc Sidak's multiple comparisons, *p* = 0.0030) during a cue‐induced seeking test. (K, L) Oxy and Sal rats travelled the same distance (K) and spent the same percentage of time in the center of the arena (L) during the OFT. (M) Oxy and Sal rats spent a similar percentage of time in the chamber with a novel conspecific rat during SoPT. (N) Oxy and Sal rats consumed the same percentage of sucrose during the SuPT. (O) Oxy and Sal rats spent the same percentage of time in the open arm during EPM. All data are presented as mean ± SEM; individual data are shown as closed circles in bar graphs. Symbols indicating significance (*) or a trend (#) are defined above for each panel. Abbreviations: abstinence day (AD), active (Act), average (avg), elevated plus maze (EPM), inactive (Ina), open field test (OFT), oxycodone (Oxy), saline (Sal), self‐administration (SA), social preference test (SoPT), somatic withdrawal (SWD), sucrose preference test (SuPT). Full statistical results for Figure 1 are provided in Table 1. See Figure S1 and Tables S1 and S2 for related analyses.

**TABLE 1:** Full statistical results for all data presented in Figures 1, 2, 3.

Figure	Statistical test	*F*, *H*, *t* or *U*	*p*
Figure 1: Early abstinence somatic withdrawal and late abstinence negative affect			
1B: SA infusions	Two‐way mixed ANOVA		
	Drug × day interaction	*F*_(9,99)_ = 14.20	< 0.0001*
	Within‐subject: Day	*F*_(1.572,17.30)_ = 9.071	0.0034*
	Between‐subject: Drug	*F*_(1,11)_ = 0.02383	0.8801
1C: SA avg infusions	Unpaired *t*‐test (Oxy, Sal)	*t*_(11)_ = 4.348	0.0012*
1D: SA nose pokes	Three‐way ANOVA		
	Drug × hole × day interaction	*F*_(9,99)_ = 4.791	< 0.0001*
	Drug × hole interaction	*F*_(1,11)_ = 0.4618	0.5108
	Drug × day interaction	*F*_(9,99)_ = 4.475	< 0.0001*
	Day × hole interaction	*F*_(9,99)_ = 2.578	0.0104*
	Between‐subject: Drug	*F*_(1,11)_ = 0.4862	0.5001
	Within‐subject: Day	*F*_(1,11)_ = 2.921	0.1155
	Within‐subject: Hole	*F*_(9,99)_ = 6.662	< 0.0001*
1E: SA avg pokes	Two‐way mixed ANOVA		
	Hole × drug interaction	*F*_(1,11)_ = 5.589	0.0375*
	Within‐subject: Hole	*F*_(1,11)_ = 3.611	0.0839^#^
	Between‐subject: Drug	*F*_(1,11)_ = 0.9043	0.3621
1F: AD1–3 weight	Two‐way mixed ANOVA		
	AD × drug interaction	*F*_(3,33)_ = 23.90	< 0.0001*
	Within‐subject: AD	*F*_(1.288,14.17)_ = 1.625	0.2284
	Between‐subject: Drug	*F*_(1,11)_ = 27.83	0.0003*
1G: AD1 SWD	Unpaired *t*‐test (Oxy, Sal)	*t*_(11)_ = 1.162	0.2700
1H: AD1 teeth chatters	Mann–Whitney test (Oxy, Sal)	*U* = 7	0.0449*
1I: AD1 WDS	Unpaired *t*‐test (Oxy, Sal)	*t*_(11)_ = 1.902	0.0836^#^
1J: AD40 seeking	Two‐way mixed ANOVA		
	Hole × drug interaction	*F*_(1,11)_ = 3.607	0.0841^#^
	Within‐subject: Hole	*F*_(1,11)_ = 14.65	0.0028*
	Between‐subject: Drug	*F*_(1,11)_ = 11.82	0.0056*
1K: OF distance	Unpaired *t*‐test (Oxy, Sal)	*t*_(11)_ = 0.4254	0.6787
1L: OF center (%)	Unpaired *t*‐test (Oxy, Sal)	*t*_(11)_ = 0.3705	0.7180
1M: SoPT (%)	Unpaired *t*‐test (Oxy, Sal)	*t*_(11)_ = 0.0554	0.9568
1N: SuPT (%)	Unpaired *t*‐test (Oxy, Sal)	*t*_(11)_ = 0.7793	0.4522
1O: EPM (%)	Unpaired *t*‐test (Oxy, Sal)	*t*_(11)_ = 1.191	0.2586
Figure 2: Early abstinence and late abstinence negative affect			
2B: SA infusions	Two‐way mixed ANOVA		
	Drug × day interaction	*F*_(9,126)_ = 8.426	< 0.0001*
	Within‐subject: Day	*F*_(3.210,44.94)_ = 8.384	0.0001*
	Between‐subject: Drug	*F*_(1,14)_ = 7.969	0.0136*
2C: SA avg infusions	Mann–Whitney test (Oxy, Sal)	*U* = 0	0.0002*
2D: SA nose pokes	Three‐way mixed ANOVA		
	Day × hole × drug interaction	*F*_(9,126)_ = 1.621	0.1159
	Hole × drug interaction	*F*_(1,14)_ = 0.6483	0.4342
	Day × drug interaction	*F*_(9,126)_ = 0.9334	0.4987
	Day × hole interaction	*F*_(9,126)_ = 2.423	0.0143*
	Between‐subject: Drug	*F*_(1,14)_ = 16.17	0.0011*
	Within‐subject: Hole	*F*_(1,14)_ = 9.174	0.0090*
	Within‐subject: Day	*F*_(9,126)_ = 3.255	0.0014*
2E: SA avg pokes	Two‐way mixed ANOVA		
	Hole × drug interaction	*F*_(1,14)_ = 1.434	0.2510
	Within‐subject: Hole	*F*_(1,14)_ = 5.689	0.0318*
	Between‐subject: Drug	*F*_(1,14)_ = 7.649	0.0152*
2F: Act pokes seeking	Two‐way mixed ANOVA		
	AD × drug interaction	*F*_(1,14)_ = 5.130	0.0399*
	Within‐subject: AD	*F*_(1,14)_ = 33.12	< 0.0001*
	Between‐subject: Drug	*F*_(1,14)_ = 10.76	0.0055^#^
2G: Incubation score	Unpaired *t*‐test (Oxy, Sal)	*t*_(14)_ = 2.265	0.0399*
2H: OF center (%)	Two‐way mixed ANOVA		
	AD × drug interaction	*F*_(1,14)_ = 1.711	0.2119
	Within‐subject: AD	*F*_(1,14)_ = 2.231	0.1575
	Between‐subject: Drug	*F*_(1,14)_ = 0.0008	0.9781
2I: OF center entries	Two‐way mixed ANOVA		
	AD × drug interaction	*F*_(1,14)_ = 0.2423	0.6302
	Within‐subject: AD	*F*_(1,14)_ = 10.92	0.0052*
	Between‐subject: Drug	*F*_(1,14)_ = 0.0937	0.7641
2J: SoPT (%)	Two‐way mixed ANOVA		
	AD × drug interaction	*F*_(1,14)_ = 0.1783	0.6793
	Within‐subject: AD	*F*_(1,14)_ = 11.98	0.0038*
	Between‐subject: Drug	*F*_(1,14)_ = 0.9635	0.3430
2K: SoPT entries	Two‐way mixed ANOVA		
	AD × drug interaction	*F*_(1,14)_ = 0.3396	0.5693
	Within‐subject: AD	*F*_(1,14)_ = 10.51	0.0059*
	Between‐subject: Drug	*F*_(1,14)_ = 0.1378	0.7161
2L: SuPT (%)	Two‐way mixed ANOVA		
	AD × drug interaction	*F*_(1,11)_ = 4.586	0.0555^#^
	Within‐subject: AD	*F*_(1,11)_ = 5.135	0.0446*
	Between‐subject: Drug	*F*_(1,11)_ = 3.850	0.0756^#^
2M: EPM (%)	Two‐way mixed ANOVA		
	AD × drug interaction	*F*_(1,14)_ = 0.0004	0.9848
	Within‐subject: AD	*F*_(1,14)_ = 8.208	0.0125*
	Between‐subject: Drug	*F*_(1,14)_ = 0.5065	0.4883
Figure 3: Housing condition and SA contribution to negative affect			
3B: OF center (%)	Mann–Whitney test (single, pair)	*U* = 25	0.5054
3C: SoPT (%)	Unpaired *t*‐test (single, pair)	*t*_(14)_ = 1.378	0.1899
3D: SuPT (%)	Unpaired *t*‐test (single, pair)	*t*_(14)_ = 2.420	0.0297*
3E: EPM center (%)	Mann–Whitney test (single, pair)	*U* = 20	0.2345
3F: OF center (%)	Kruskal–Wallis test (single, Sal, Oxy)	*H* = 0.4915	0.7821
3G: SoPT (%)	One‐way ANOVA (single, Sal, Oxy)	*F*_(2,34)_ = 1.906	0.1642
3H: SuPT (%)	One‐way ANOVA (single, Sal, Oxy)	*F*_(2,31)_ = 5.204	0.0113*
3I: EPM (%)	Kruskal–Wallis test (single, Sal, Oxy)	*H* = 8.145	0.0170*

*Note:* The table outlines the statistical test used, results and *p*‐values for data included in Figures 1, 2, 3.

Abbreviations: abstinence day (AD), active (Act), average (avg), elevated plus maze (EPM), inactive (Ina), open field (OF), oxycodone (Oxy), saline (Sal), self‐administration (SA), social preference test (SoPT), somatic withdrawal (SWD), sucrose preference test (SuPT).

* *p* < 0.05.

^#^ *p* < 0.1.

Male and female rats underwent extended‐access oxycodone or saline SA. The number of infusions across the 10 days of training is shown in Figure 1B (drug × day interaction, *F*_(9,99)_ = 14.20, *p* < 0.0001); nose pokes are shown in Figure 1D (drug × hole × day interaction, *F*_(9,99)_ = 4.791, *p* < 0.0001). Oxycodone rats took more infusions than saline rats (Figure 1C; unpaired *t*‐test, *t*_(11)_ = 4.348, *p* = 0.0012) and poked more in the active than inactive hole (Figure 1E; hole × drug interaction, *F*_(1,11)_ = 5.589, *p* = 0.0375) during the last 3 days of SA, indicating successful acquisition of oxycodone SA.

Weight loss typically occurs during non‐contingent escalating dose opioid regimens and persists during early abstinence (e.g., [11]). We recorded weight before SA training and on AD1–3. When data were normalized to weight before SA, oxycodone rats lost weight while saline rats gained weight over AD1–3 (Figure 1F; AD × drug interaction, *F*_(3,33)_ = 23.90, *p* < 0.0001), but there were no significant differences in absolute weight between the groups (Figure S1A). After SA training, we assessed grooming, wet dog shakes, teeth chatters, jumps and digging individually on AD1, AD2 and AD3 (Figure S1B–G). Analysis of this 3‐day period revealed that oxycodone rats exhibited more teeth chatters (main effect of drug, *F*_(1,11)_ = 6.803, *p* = 0.0243) and a trend towards more wet dog shakes (drug × AD interaction, *F*_(2,22)_ = 3.099, *p* = 0.0652) (Figure S1C,D). We then restricted our comparison of saline and oxycodone rats to AD1, a time when spontaneous SWD symptoms peak after non‐contingent opioids (e.g., [12, 16]). We found a significant difference between saline and oxycodone rats for teeth chatters (Mann–Whitney test, *U* = 7, *p* = 0.0449) and a trend towards significance for wet dog shakes (unpaired *t*‐test, *t*_(11)_ = 1.902, *p* = 0.0836) (Figure 1H,I) but not for other symptoms (*p* > 0.05, data not shown). The SWD score (derived by adding up the incidence of each symptom for each animal on AD1; see Section 2) exhibited by oxycodone rats did not differ significantly from the score for saline rats (Figure 1G). Together, these results suggest that our SA regimen produced a modest degree of physical dependence with the most pronounced symptoms occurring on AD1. It is possible that variability among rats is related to individual differences in the number of oxycodone infusions taken during SA training. We tested this by evaluating correlations between total oxycodone consumed (mg/kg) over all 10 days of SA and the weight change, total SWD score and each individually scored behaviour for SWD on AD1 (Table S2). We found a trend towards a significant positive correlation between oxycodone intake and wet dog shakes on AD1 (Pearson's *r* = 0.7054, *p* = 0.077); no other correlations approached significance.

Starting on AD40, rats received a cue‐induced seeking test followed by tests of negative affect (OFT, SoPT, SuPT and EPM), with the order of tests selected to minimize impact of earlier tests on later tests (Figure 1A). Oxycodone rats showed greater cue‐induced seeking than saline rats (Figure 1J; main effect of drug, *F*_(1,11)_ = 11.82, *p* < 0.0056), but subsequent tests for anxiety‐ or depressive‐like behaviour failed to reveal group differences. Specifically, there were no differences in distance travelled or time in the center of an open field (Figure 1K,L), preference for a stranger conspecific (Figure 1M), sucrose preference (Figure 1N) or time in open arms of the EPM (Figure 1O) (unpaired *t*‐tests, *p* > 0.05). Additional measures made during these tests are shown in Figure S1H–L.

Overall, these results suggest that our oxycodone SA regimen elicits some SWD signs in early abstinence but does not result in detectable negative affective states after protracted abstinence compared to rats that self‐administered saline.

### 3.2 Comparison of Behaviours Indicative of Negative Affect in Early and Late Abstinence From Saline or Oxycodone SA

Although studies in Figure 1 revealed no difference between saline and oxycodone rats in negative affect tests after protracted abstinence, we generated a second cohort of saline and oxycodone rats to determine if changes could be detected within each group as a function of abstinence duration. These rats received seeking tests and negative affect tests in both early abstinence (AD1–7) and protracted abstinence (AD40–46) (Figure 2A). Full statistics for all results in Figure 2 are shown in Table 1. As in the first cohort, oxycodone rats took more infusions than saline rats over 10 days of SA (Figure 2B; drug × day interaction, *F*_(9,126)_ = 8.426, *p* < 0.0001), had greater average infusions over the last 3 days of SA (Figure 2C; Mann–Whitney test, *U* = 0, *p* = 0.0002) and discriminated between active and inactive holes (Figure 2E; main effect of hole, *F*_(1,14)_ = 5.689, *p* = 0.0318); nose pokes during each day of SA are presented in Figure 2D (main effect of hole, *F*_(1,14)_ = 9.174, *p* = 0.0090). During seeking tests, responses in the previously active hole were significantly greater on AD40 than AD1 for oxycodone rats (Figure 2F; AD × drug interaction, *F*_(1,14)_ = 5.130, *p* = 0.0399), whereas inactive hole responses (Figure S2A) and active pokes by saline rats did not show significant time dependency. Accordingly, the incubation score (AD40 − AD1 active pokes) was significantly greater for oxycodone rats (Figure 2G; unpaired *t*‐test, *t*_(14)_ = 2.265, *p* = 0.0399). These data indicate that oxycodone rats demonstrated incubation of craving on AD40. To assess consistency of oxycodone intake between the cohort of oxycodone rats depicted in Figure 1 and those in Figure 2, we analysed total oxycodone consumption (mg/kg) between the two cohorts across all 10 days of SA and found no statistically significant difference between the groups (Mann–Whitney test, *U* = 32, *p* = 0.8125; median consumption [mg/kg]: Figure 1 = 137.9, Figure 2 = 123.4). This indicates comparable oxycodone intake in the two cohorts.

**FIGURE 2:**
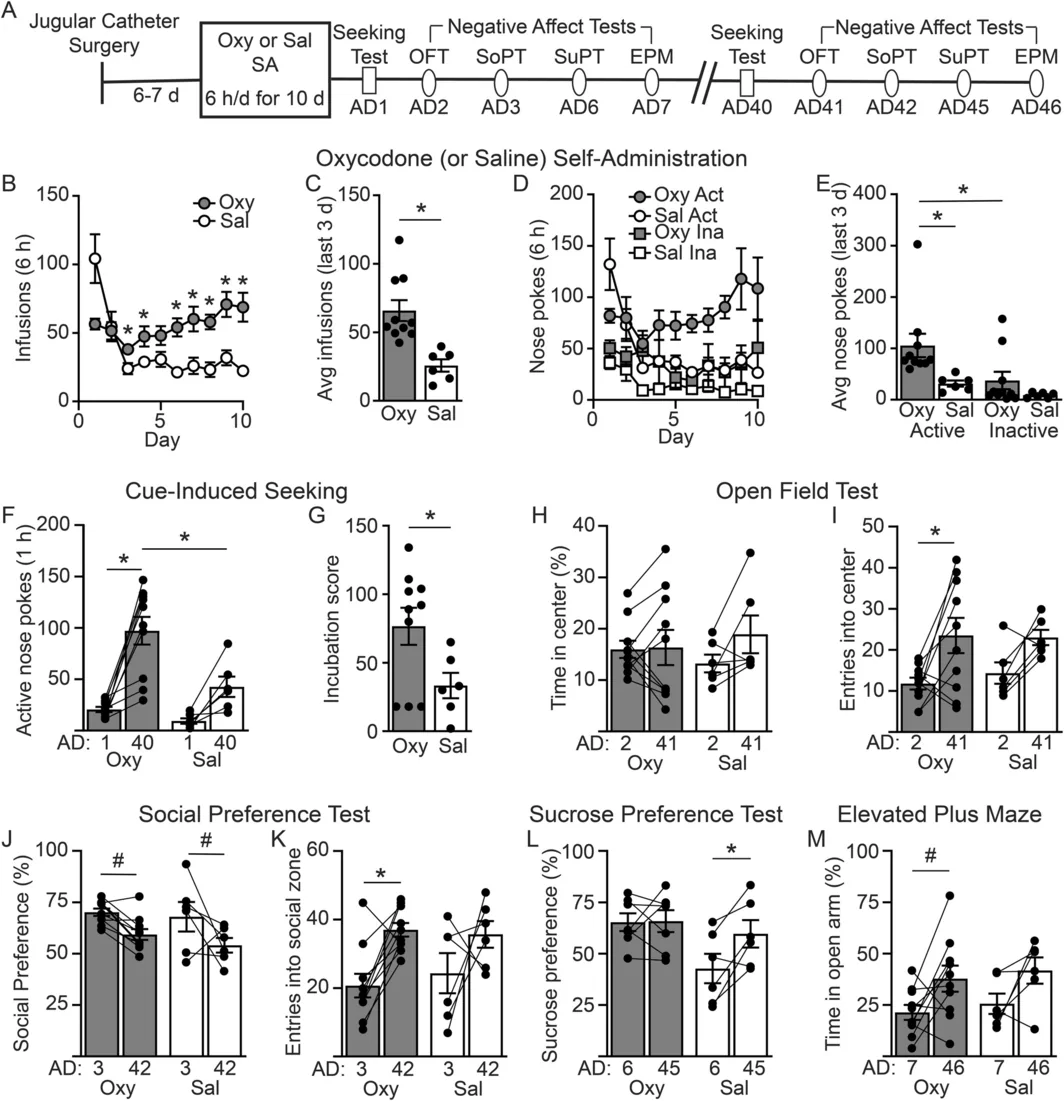
Negative affect during incubation of craving following oxycodone self‐administration (SA). (A) Rats received jugular catheters and recovered for 6–7 days. Rats self‐administered oxycodone (Oxy; 0.1 mg/kg/infusion; *n* = 10) or saline (Sal; *n* = 6) for 6 h/day for 10 days. Rats then underwent a series of behaviour tests in early abstinence: cue‐induced seeking tests (1 h) on abstinence day (AD)1, open field test (OFT) on AD2, social preference test (SoPT) on AD3, sucrose habituation on AD4–5, sucrose preference test (SuPT) on AD6 and elevated plus maze (EPM) on AD7. Rats remained in their home cage until late abstinence and then underwent the same sequence of behaviour tests on AD40–46. (B) The number of infusions of Oxy or Sal self‐administered in each 6‐h session across the 10 days of SA. Oxy rats took a larger number of infusions than Sal rats on Days 3–4 and 6–10 (post hoc Sidak's multiple comparisons test, *p* < 0.05 for each day). (C) During the last 3 days of SA, Oxy rats took a significantly higher number of infusions than Sal rats (Mann–Whitney test, *U* = 0, *p* = 0.0002). (D) Nose pokes in the active (Act) and inactive (Ina) hole in rats that self‐administered Oxy or Sal. (E) During the last 3 days of SA, Oxy rats poked more in the active (Act) hole than the inactive (Ina) hole (post hoc Sidak's multiple comparisons test, *p* = 0.0220), an effect not observed in Sal rats. Oxy rats poked more in the active hole than Sal rats (post hoc Sidak's multiple comparisons test, *p* = 0.0186). (F) The number of active pokes during cue‐induced seeking tests on AD1 and AD40. Oxy rats poked more in the active hole on AD40 than on AD1 (post hoc uncorrected Fisher's LSD, *p* < 0.0001) and compared to Sal rats (post hoc uncorrected Fisher's LSD, *p* = 0.0005). (G) The incubation score (AD40 Act − AD1 Act) was significantly higher in Oxy rats compared to Sal rats (unpaired *t*‐test, *p* = 0.0399). (H) Oxy and Sal rats spent the same amount of time in the center of the open field on AD2 and AD41. (I) Oxy rats entered the center of the open field more times on AD41 compared to AD1 (post hoc Sidak's multiple comparisons test, *p* = 0.0156). (J) Oxy and Sal rats trended towards a reduction in time spent exploring a novel conspecific from AD3 to AD42 (post hoc Sidak's multiple comparisons test AD3 vs. AD42, Oxy: *p* = 0.0521, Sal: *p* = 0.0547). (K) Oxy rats entered the zone around the novel conspecific more on AD42 compared to AD3 (post hoc Sidak's multiple comparisons test, *p* = 0.0149), whereas this was not significant for Sal rats. (L) Sal rats increased sucrose preference from AD6 to AD45 (post hoc Sidak's multiple comparisons, *p* = 0.0239), whereas Oxy rats did not change. (M) Oxy rats trended towards an increase in the amount of time spent in the open arm from AD7 to AD46 (post hoc Sidak's multiple comparisons test, *p* = 0.0661). Symbols indicating significance (*) or a trend (#) are defined above for each panel. Abbreviations: abstinence day (AD), active (Act), average (avg), elevated plus maze (EPM), inactive (Ina), open field test (OFT), oxycodone (Oxy), saline (Sal), self‐administration (SA), social preference test (SoPT), sucrose preference test (SuPT). Full statistical results for Figure 2 are provided in Table 1. See Figure S2 and Table S1 for additional analyses.

Interestingly, both saline and oxycodone rats showed some time‐dependent changes in negative affect tests. Commonly reported metrics are shown in Figure 2, whereas Figure S2 shows additional supporting analyses (full statistics for Figure S2 in Table S1). In the OFT, total distance travelled (Figure S2B) and time in the center (Figure 2H) did not differ as a function of AD or drug group. However, entries into the center of the open field (Figure 2I) increased from AD2 to AD41 (main effect of AD, *F*_(1,14)_ = 10.92, *p* = 0.0052), with post hoc tests indicating a significant effect for oxycodone rats (*p* = 0.0156). In the SoPT, time spent with the novel conspecific rat (Figure 2J) showed a significant main effect of AD (*F*_(1,14)_ = 11.98, *p* = 0.0038), with post hoc tests showing trends towards lower preference on AD42 for both saline and oxycodone rats compared to AD3 (*p* = 0.0547 and 0.0521, respectively). Interestingly, entries into the social zone (Figure 2K) tended to increase in late abstinence in oxycodone rats. In the SuPT, saline rats showed greater preference on AD45 versus AD6, whereas oxycodone rats did not change (Figure 2L; main effect of AD, *F*_(1,11)_ = 5.135, *p* = 0.0446). In EPM tests, while entries into the open arm did not differ by time or group (Figure S2D), time spent in the open arm (Figure 2M) showed a main effect of AD (*F*_(1,14)_ = 8.208, *p* = 0.0125), with post hoc tests showing that oxycodone rats trended towards more time in the open arm (*p* = 0.0661) on AD46.

These results suggest that modest time‐dependent changes occur in both groups, although they are not suggestive of intensification of negative affect in oxycodone rats as abstinence progresses. Thus, oxycodone rats show less anxiety‐like behaviour in late abstinence in some tests (center entries in OFT and open arm time in EPM), although saline rats showed similar trends (Figure 2I,M). Both oxycodone and saline rats trend towards lower social preference in late abstinence (Figure 2J), and saline rats show a small increase in sucrose preference in late abstinence (Figure 2L). However, when saline and oxycodone rats are compared in late abstinence, data in both Figures 1 and 2 indicate no robust differences between the groups.

### 3.3 Comparison of Single‐ and Pair‐Housed Rats

During incubation experiments, rats are single‐housed for ~9 weeks (Figures 1A and 2A). To determine if single housing for this prolonged period influences measures of negative affect after incubation, a control experiment was conducted to isolate this variable by comparing drug‐naïve rats that were single‐ or pair‐housed for a similar length of time (56–61 days; Figure 3A) using OFT, SoPT, SuPT and EPM (Figure 3B–E and Table 1). The only group difference identified was lower sucrose preference in pair‐housed rats (Figure 3D; unpaired *t*‐test, *t*_(14)_ = 2.42, *p* = 0.0297). Additional metrics are shown in Figure S3 (total distance travelled and entries into center for OFT; time in social zone and entries into social zone for SoPT; amount consumed for SuPT; entries into open arm for EPM; statistical results in Table S1). Figure 3F–I shows data from the drug‐naïve, single‐housed rats replotted alongside the oxycodone and saline forced abstinence groups (combined from Figures 1 and 2). No group differences were found for time in the center of the open field or social preference (Figure 3F,G), but single‐housed, drug‐naïve rats showed greater sucrose preference (Figure 3H; one‐way ANOVA, *F*_(2,31)_ = 5.204, *p* = 0.0113) and less time in the open arm of the EPM (Figure 3I; Kruskal–Wallis test, *H* = 8.145, *p* = 0.0170) compared to the saline and oxycodone groups (which did not differ from each other).

**FIGURE 3:**
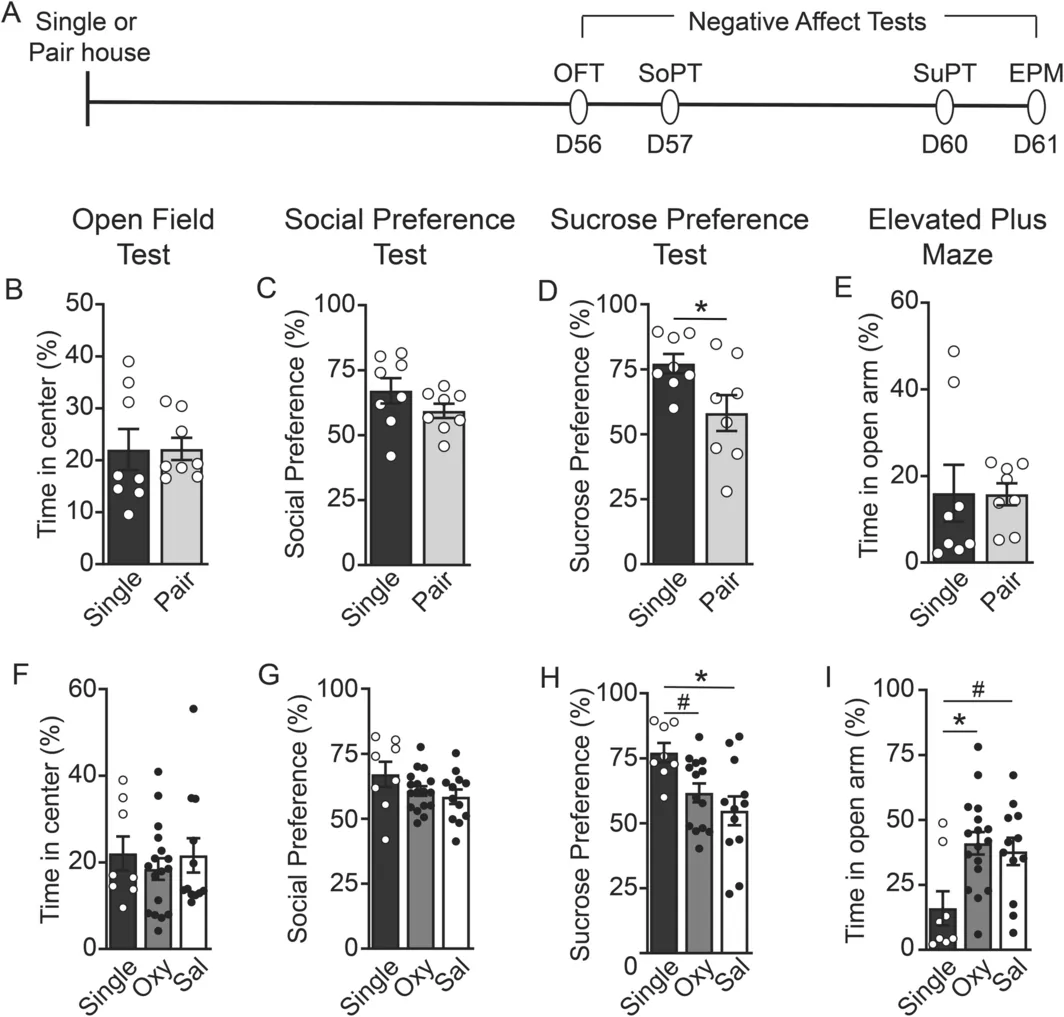
Effects of housing condition and self‐administration training on assays measuring negative affect. (A) A cohort of naïve rats was single‐ or pair‐housed for 8 weeks. Rats then underwent open field test (OFT) on Day 56, social preference test (SoPT) on Day 57, sucrose preference test (SuPT) on Days 58–60 and elevated plus maze (EPM) on Day 61. (B) Single‐ and pair‐housed rats spent the same amount of time in the center of the open field. (C) The preference for a novel rat was not different between single‐ and pair‐housed rats. (D) Pair‐housed rats exhibited a lower sucrose preference than single‐housed rats (unpaired *t*‐test, *p* = 0.0399). (E) Housing status did not change the amount of time spent in the open arm of the EPM. (F) Rats that self‐administered oxycodone (Oxy) or saline (Sal) spent a similar amount of time in the center of the open field as single‐housed, drug‐naïve rats. (G) Self‐administration did not alter the time spent exploring a novel rat compared to single‐housed, drug‐naïve rats. (H) While Sal and Oxy rats did not demonstrate differences in sucrose preference, single‐housed, drug‐naïve rats had higher sucrose preference compared to Sal rats (post hoc Tukey's multiple comparisons test, *p* = 0.0086) and trended towards the same effect relative to Oxy rats (*p* = 0.0736). (I) While Sal and Oxy rats did not demonstrate differences in the time spent in the open arm of the EPM, single‐housed, drug‐naïve rats spent less time in the open arm of the EPM compared to Oxy rats (post hoc Dunn's multiple comparisons test, *p* = 0.0181) and trended towards the same effect relative to Sal rats (*p* = 0.0559). Symbols indicating significance (*) or a trend (#) are defined above for each panel. Abbreviations: elevated plus maze (EPM), open field test (OFT), oxycodone (Oxy), saline (Sal), social preference test (SoPT), sucrose preference test (SuPT). Full statistical results are provided in Table 1. See Figure S3 and Table S1 for additional analyses.

Together, these results indicate that single housing is not sufficient to elicit negative affective states under our conditions (see Section 4.4 for more discussion). The increased anhedonia‐like behaviour (SuPT) and reduced anxiety‐like behaviour (EPM) in saline and oxycodone SA rats after protracted abstinence compared to drug‐naïve rats single‐housed for a similar duration may be related to a history of surgery and operant training in the former groups.

## 4 Discussion

### 4.1 Overview and Major Conclusions

The major goal was to measure negative affective states over the course of protracted abstinence from an oxycodone SA regimen that elicits incubation of cue‐induced oxycodone seeking. Our first experiment compared rats after oxycodone or saline SA. Although oxycodone rats exhibited spontaneous SWD signs on AD1, incubation of oxycodone craving was not accompanied by anxiety‐ or depressive‐like behaviour as compared to control rats that self‐administered saline (measures made on AD40+). In our second experiment, we performed a within‐subject study in which the same measures of negative affect were performed in early (AD2–7) and late abstinence (AD41–46). While we identified modest changes in affective states within each treatment group over time, these changes did not support the idea that progressive increases in negative affective state accompany the incubation of craving. Details on each experiment, as well as a control study assessing the potential impact of single housing on our results, are described below.

Our findings are significant because they indicate that rats exhibiting incubation are not seeking oxycodone to relieve an aversive state that persists into protracted abstinence. An alternative explanation is an increase in salience of the oxycodone‐paired cue over the course of protracted abstinence. Consistent with this, adaptations associated with the regulation of cue reactivity have been implicated in oxycodone incubation, such as strengthening of glutamate synapses in the NAc [21, 30, 31]. Adaptations in other reward‐related pathways (e.g., [22, 23]) and regulatory mechanisms (e.g., gene expression) have also been reported after incubation of craving for oxycodone and other opioids [3].

### 4.2 Negative Affective States Do Not Emerge in Rats Exhibiting Incubation of Oxycodone Craving

It is well established that people recovering from OUD often experience persistent negative affective states that contribute to relapse and that negative affective states are observed in rodents after non‐contingent escalating dose regimens of opioids and can sometimes intensify over several weeks of withdrawal (see Section 1). Far less is known about whether negative affective states occur after protracted abstinence from opioid SA. In our first cohort of rats, we performed OFT, SoPT, SuPT and EPM after protracted withdrawal (AD40+) from oxycodone SA or saline SA. We observed no differences between the oxycodone and saline groups. No other studies have assessed negative affect after protracted oxycodone withdrawal, but two have done so after heroin SA. First, a study of incubation of heroin craving measured ultrasonic vocalizations (USVs) during AD1 and AD30 seeking tests; this study found an increase in 50‐kHz USVs (which indicate a rewarding situation) on WD30 in males but not females but detected very few 22‐kHz USVs (which indicate a negative state) in any group [32]. Relatively low 22‐kHz USVs after heroin incubation are consistent with our findings of no obvious negative affective states after oxycodone incubation. Second, a recent study found that male and female mice tested 14 days after discontinuing heroin SA spent significantly less time interacting with a social stimulus (age‐ and sex‐matched mouse) compared to saline controls [33]. In mice with lower than median heroin seeking on AD13, there was a significant negative correlation between social interaction time and heroin seeking [33]. In this prior study, only the social stimulus was present during the test. We used a different social interaction test in which rats could interact with a stranger conspecific or an empty container during the test, and results are reported as the percentage of time interacting with the stranger conspecific (see Section 2), and we found no difference between saline and oxycodone rats (Figures 1 and 2). The use of different test procedures, as well as different opioids and species, may underlie the different results.

Our first experiment involved a between‐group (saline/oxycodone) comparison at a single range of abstinence times (AD41–46). We therefore generated a second cohort of saline and oxycodone SA rats for a within‐subject study comparing early (AD2–7) and late (AD41–46) abstinence. As detailed in Section 3 and in Figure 2, we did find some time‐dependent changes within each treatment group. Overall, however, the measured behaviours in saline rats remained stable across abstinence with only a modest reduction in social preference and an increase in sucrose preference in late abstinence, whereas oxycodone rats showed a similar reduction in social preference but also exhibited OFT and EPM behaviours consistent with reduced anxiety‐like behaviour in late versus early abstinence. It is worth noting that while many studies report reduced time in the open arm of the EPM after escalating morphine doses, including two conducted after 1–4 weeks of withdrawal [16, 34], one study found the opposite result (similar to ours) in male mice 6 weeks after morphine withdrawal and interpreted this as impulsive behaviour [35], which has been observed in human opioid users (e.g., [36]). Overall, our results do not support the idea that negative affective states intensify as abstinence progresses and contribute to the incubation of oxycodone craving through a negative reinforcement mechanism. Likewise, a study of heroin incubation discussed in the previous section found no change in 22‐kHz USVs (which indicate a negative state) over the course of heroin incubation [32].

The absence of differences in negative affect between saline and oxycodone rats in early abstinence is interesting to consider in light of prior studies. Focusing first on oxycodone, tests performed in the first 24 h after an oxycodone SA session revealed irritability [37, 38] and elevated brain reward threshold [39], but these studies used a higher oxycodone dose (0.15 mg/kg/infusion) and a longer daily session (12 h). A 12‐h session was also used in a study reporting potentiated acoustic startle, interpreted as anxiety‐like behaviour, after 10–20 h of withdrawal from heroin SA [40].

### 4.3 SWD Signs in Early Abstinence From Oxycodone SA

In our first cohort of rats, we quantified spontaneous SWD signs on AD1–3 following saline or oxycodone SA. While grooming, jumps and digging did not differ between the saline and oxycodone groups, the oxycodone rats showed significantly more teeth chatters and a trend towards more wet dog shakes on AD1. It is significant that these particular behaviours were exhibited; both are classical signs of opioid withdrawal, and although they reflect dysfunction of different systems within the nervous system, both are considered strong indicators of the magnitude of acute distress and dependence [41]. Interestingly, not all oxycodone rats exhibited these behaviours, but across all rats there was a near‐significant positive correlation between teeth chatters and oxycodone intake during SA training. Oxycodone rats also exhibited reduced body weight on AD1–3 relative to their pre‐SA weight, consistent with prior reports using non‐contingent morphine dosing [11]. Together, these results indicate that our oxycodone SA regimen produced a modest degree of physical dependence. Consistent with this, a prior study using a short‐access oxycodone SA regimen observed SWD signs 0 and 22 h after the last session, but their magnitude is difficult to assess because vehicle controls were not included [26]. Another study found significant SWD signs in oxycodone rats compared to vehicle rats 24 h after discontinuing an extended‐access regimen that included more sessions than ours (22 vs. 10) [31]. It would be interesting in the future to assess the magnitude of physical dependence produced by our regimen using naloxone‐precipitated withdrawal.

Two factors likely contribute to modest SWD signs and absence of detectable negative affect following our SA regimen as compared to non‐contingent escalating dose regimens (see Section 1). First, volitional drug intake may be less aversive or engage distinct motivational or neurobiological processes compared to experimenter‐administered drugs. Second, total opioid exposure is lower in SA studies. In the present study, rats (300–400 g) self‐administered 15–20 mg of oxycodone over 10 days, whereas non‐contingent escalating regimens deliver substantially higher doses over shorter timeframes (e.g., ~63 mg of oxycodone in a 20–25‐g mouse over 9 days [42]), resulting in markedly greater exposure relative to body mass.

### 4.4 Single Housing Alone Does Not Necessarily Elicit Negative Affective States

In incubation experiments, rats are typically single‐housed following jugular catheter surgery, with single housing continuing throughout SA training and protracted abstinence. We wondered whether single housing might have influenced measures of negative affect performed after saline or oxycodone SA. To isolate this variable, drug‐naïve rats were either single‐ or pair‐housed for a period of time equivalent to post‐surgical isolation during an incubation experiment (8+ weeks) prior to assessing negative affective states. We found that housing condition did not significantly alter performance in OFT, SoPT or EPM, although pair‐housed rats showed increased anhedonia‐like behaviour (SuPT). Notably, when compared to the single‐housed, drug‐naïve rats, those rats that underwent either oxycodone or saline SA exhibited increased anhedonia‐like behaviour (SuPT) and reduced anxiety‐like behaviour (EPM). Overall, these results suggest that prior surgical and operant training experience had a greater impact on affective state than housing conditions. Prior work has shown that the effects of social isolation on affective states are variable, with minimal impact on locomotion and unconditioned anxiety‐like behaviours (i.e., OFT and EPM) and moderate effects on SuPT and SoPT [43, 44]. Furthermore, the effects of social isolation depend on factors such as sex, strain and comparator housing conditions. In contrast to prior studies that typically initiate isolation during adolescence (e.g., [43, 44]), our study examined isolation beginning in young adulthood for a duration aligned with incubation procedures. These differences in developmental timing and protocol are likely to influence the emergence of affect‐related behaviours. Collectively, the available evidence suggests that single‐housing of rats is not sufficient to induce negative affect‐like behaviour, particularly in standard colony environments where rats maintain auditory, visual and olfactory interactions with conspecifics in the housing room. We also note that single versus pair housing did not affect incubation of heroin craving in either sex [45].

### 4.5 Sex as a Biological Variable

We used males and females in all experiments and did not observe obvious differences, consistent with our prior study of oxycodone incubation [21] and many prior studies of opioid incubation (for review, see [3, 46]). Because of these prior results and because sex was not our primary independent variable of interest, our individual experiments were powered to detect effects of treatment but not sex. We acknowledge that this is a limitation of our study given evidence for sex differences in some relevant rodent studies (summarized below) and in human studies [47, 48]. It is important to note, however, that the only paper examining behaviours related to negative affect after oxycodone SA found no sex differences in these behaviours [37]. Below, we summarize data from our lab and others on the influence of sex on oxycodone SA, followed by consideration of the influence of sex on oxycodone incubation.

Regarding SA training, in the present study we did not observe sex differences in active pokes, inactive pokes or oxycodone infusions when we analysed data from all rats across experiments (nine females and eight males; statistics are shown in Table S3). In a prior study using the same wild‐type Long‐Evans rats and oxycodone regimen (22 females and 36 males), we also observed no sex differences in active pokes, inactive pokes or oxycodone infusions; furthermore, no significant sex differences for these measures were found for transgenic rats on a Long‐Evans background (total of 15 males and 16 females) [21]. Our data are consistent with several other oxycodone incubation studies using long‐access SA regimens [24, 31, 38], although sex differences in some parameters were found in other studies [25, 37, 49, 50].

Regarding incubation of craving, in the present study, we did not observe a sex difference in the incubation score (Table S3). Likewise, in our previous study [21], we compared active hole responses between male and female oxycodone rats during the AD1, AD15 and AD30 seeking tests using a two‐way ANOVA with sex and AD as between‐subject factors and found a significant main effect of AD but not sex; we also found no sex difference in the incubation score. Many prior studies from other groups have shown incubation of oxycodone seeking in one sex or the other (see [21]; limits on reference number prevent us from providing a full list here), but only two studies other than our own have compared males and females. One found significant incubation in females but not males on AD14 [31], and the other observed higher seeking on AD14 in females [50]. Finally, in two studies that assessed the impact of the estrous cycle on incubated oxycodone seeking, one found no effect on AD15 [23], and the other found that females in estrus at the time of an AD43–45 seeking test did not exhibit incubation, whereas non‐estrus females did [24].

In conclusion, work from our lab has shown that both sexes exhibit incubation, consistent with most reports in the literature. Furthermore, while our studies of negative affective states were not powered to detect sex differences, we did not observe obvious effects of sex, consistent with results of the only other study that examined behaviours related to negative affect after oxycodone SA [37].

## 5 Conclusions

We conclude that negative affective states do not occur alongside incubation of craving using a standard oxycodone incubation regimen, probably due in part to lower drug intake during our SA regimen compared to non‐contingent escalating dose opioid regimens typically used to study SWD signs and negative affective behaviour. This does not diminish the utility of the incubation model for studying cue‐induced oxycodone craving and its neurobiological basis. Furthermore, we note that the level of oxycodone intake in our paradigm approximates clinically relevant exposure associated with pain management in humans (see Table S4), raising the possibility that clinically relevant patterns of opioid intake may elicit persistent adaptations that increase reactivity to opioid‐related cues.

## Author Contributions

Conception and study design: AMW, KAM and MEW. Data acquisition: AMW, AG, ABK, JGW, HMK and MMB. Analysis and/or interpretation of data: AMW and MEW. Drafting of the manuscript: AMW and MEW. Obtained funding: MEW. All authors reviewed and approved the manuscript.

## Funding

This study was supported by USPHS Grant DA059601 (MEW). KAM was supported by a predoctoral National Science Foundation (NSF) fellowship, and HMK was supported by USPHS F31 DA064365. KAM and HMK also received ARCS Foundation Oregon Scholarships.

## Ethics Statement

Animal studies were approved by the Institutional Animal Care and Use Committee at Oregon Health & Science University in accordance with the NIH Guide for the Care and Use of Laboratory Animals.

## Conflicts of Interest

The authors declare no conflicts of interest.

## Supporting information


**Figure S1:** Additional somatic withdrawal, open field, social preference test, sucrose preference test, and elevated plus maze data associated with Figure 1.
**Figure S2:** Additional cue‐induced seeking, open field, social preference test, sucrose preference test, and elevated plus maze data associated with Figure 2.
**Figure S3:** Additional open field test, social preference test, sucrose preference test, and elevated plus maze data associated with Figure 3.
**Table S1:** Full statistical results for all data presented in supplementary figures.
**Table S2:** Correlation analysis of oxycodone (Oxy) consumption and spontaneous somatic withdrawal signs on abstinence day 1.
**Table S3:** Comparison of male and female rats during oxycodone self‐administration and seeking tests.
**Table S4:** Rat equivalent daily dosing of oxycodone (Oxy) relative to human dosing for pain management.

## Data Availability

Data are available upon request from the communicating author.
