# Supplementary material for: Negative Affective States Are Not Detected in Rats Following an Intravenous Self‐Administration Regimen Leading to Incubation of Oxycodone Craving
Source: Addict Biol. 2026 Nov 2;31(11):e70196. doi: 10.1111/adb.70196 (PMC13727899; doi:10.1111/adb.70196)
Supplement: Supplementary file 1 [file ADB-31-e70196-s001.docx]

**Negative affective states are not detected in rats following an intravenous self-administration regimen leading to incubation of oxycodone craving**

Amanda M. Wunsch, Kimberley A. Mount, Asia Guzman, Alex B. Kawa, Jonathan G. Westlake, Hayley M. Kuhn, Madelyn M. Beutler, Marina E. Wolf

**Supplemental Material (Figures S1-S3, Tables S1-S4)**

**Figure S1. Additional somatic withdrawal, open field, social preference test, sucrose preference test, and elevated plus maze data associated with Figure 1.** Full statistical analysis for data presented in this figure is shown in Table S1. A) The weight of rats that self-administered Oxy did not differ from rats that self-administered Sal before SA or on AD1-3, although Oxy but not Sal rats showed a statistically significant reduction in weight over this period compared to their pre-SA weight (see Fig. 1F). B) The somatic withdrawal score did not differ between Oxy and Sal rats on AD1-3. C) Oxy rats exhibited more teeth chatters than Sal rats on AD1-3 (main effect of drug, *p* = 0.0243). Selective analysis of AD1 also revealed a significant effect as shown in main text Fig. 1H. D) There was no significant difference in the number of wet dog shakes between Oxy and Sal rats when AD1-3 was analyzed, although selective analysis of AD1 revealed a trend as shown in main text Fig. 1I. E) Oxy and Sal rats exhibited a similar number of grooming bouts on AD1-3. F) Oxy and Sal rats did not differ significantly in the number of jumps on AD1-3. G) Oxy SA did not alter the number of digging bouts on AD1-3 compared to Sal rats. H) Oxy and Sal rats entered the center of the open field the same number of times. I) Prior to the social preference test, Oxy and Sal rats spent the same amount of time in the zone where the novel conspecific was placed. J) During the social preference test session, Oxy and Sal rats made the same number of entries into the chamber containing a novel conspecific as the chamber without a rat. K) The amount of sucrose and water consumed (ml/kg) was the same between Oxy and Sal rats. L) The number of entries into the open arm was the same between Oxy and Sal rats. Abbreviations: abstinence day (AD), oxycodone (Oxy), saline (Sal).

**Figure S2. Additional cue-induced seeking, open field, social preference test, sucrose preference test, and elevated plus maze data associated with Figure 2.** Full statistical analysis for data presented in this figure is shown in Table S1. A) The number of inactive pokes during cue-induced seeking tests on abstinence day (AD) 1 and AD40. Both oxycodone (Oxy) and saline (Sal) rats show an increase in poking in the inactive hole from AD1 to AD40 (post hoc Sidak’s multiple comparisons test, Oxy p=0.0071, Sal p=0.0428). B) Both Oxy and Sal rats travelled the same distance in the open field on AD2 and AD41. C) Prior to testing, Oxy and Sal rats did not exhibit a preference for the zone where the novel conspecific was placed. D) Neither group showed a significant difference in entries into the open arm on AD7 versus AD46. *p<0.05. Abbreviations: abstinence day (AD), oxycodone (Oxy), saline (Sal).

**Figure S3. Additional open field test, social preference test, sucrose preference test, and elevated plus maze data associated with Figure 3.** Full statistical analysis for data presented in this figure is shown in Table S1. A-B) The total distance traveled (A) and number of entries in the center (B) of the open field were not different between single-housed or pair-housed rats. C-D) Single-housed and pair-housed rats did not exhibit a preference for either side of the social assay chamber during habituation (C) and entered the zone containing the novel conspecific rat the same number of times (D) during the social preference test. E) The amount of sucrose consumed during the sucrose preference test was the same between single-housed and pair-housed rats. F) Single-housed and pair-housed rats entered the open arm on the elevated plus maze the same number of times.

**Table S1. Full statistical results for all data presented in Supplementary Figures.**

| **Figure** | **Statistical Test** | **F, t, or U** | **p-value** |
| --- | --- | --- | --- |
| **Figure S1:** Early abstinence somatic withdrawal and late abstinence negative affect | | | |
| S1A: AD1-3 weight | Two Way Mixed ANOVA |  |  |
|  | AD x Drug interaction | F_(3,33)_=12.65 | <0.0001***** |
|  | Within subject: AD | F_(1.362,14.98)_=0.9834 | 0.3650 |
|  | Between subject: Drug | F_(1,11)_=0.02587 | 0.8751 |
| S1B: AD1-3 SWD | Two Way Mixed ANOVA | | |
|  | AD x Drug interaction | F_(2,22)_=1.597 | 0.2252 |
|  | Within subject: AD | F_(1.610,17.71)_=1.867 | 0.1876 |
|  | Between subject: Drug | F_(1,11)_=0.2444 | 0.6308 |
| S1C: AD1-3 teeth chatters | Two Way Mixed ANOVA |  |  |
|  | AD x Drug interaction | F_(2,22)_=0.8171 | 0.4547 |
|  | Within subject: AD | F_(1.880,20.68)_=1.198 | 0.3195 |
|  | Between subject: Drug | F_(1,11)_=6.803 | 0.0243* |
| S1D: AD1-3 WDS | Two Way Mixed ANOVA |  |  |
|  | AD x Drug interaction | F_(2,22)_=3.099 | 0.0652# |
|  | Within subject: AD | F_(1.484,16.33)_=0.7284 | 0.4585 |
|  | Between subject: Drug | F_(1,11)_=0.5939 | 0.4571 |
| S1E: AD1-3 grooming | Between subject: Drug | F_(1,11)_=6.803 | 0.0243***** |
|  | AD x Drug interaction | F_(2,22)_=0.02418 | 0.9761 |
|  | Within subject: AD | F_(1.545,17.00)_=2.702 | 0.1058 |
|  | Between subject: Drug | F_(1,11)_=0.03902 | 0.8470 |
| S1F: AD1-3 jumps | Two Way Mixed ANOVA |  |  |
|  | AD x Drug interaction | F_(2,22)_=1.405 | 0.2666 |
|  | Within subject: AD | F_(1.123,12.35)_=0.8247 | 0.3950 |
|  | Between subject: Drug | F_(1,11)_=2.196 | 0.1664 |
| S1G: AD1-3 digging | Two Way Mixed ANOVA |  |  |
|  | AD x Drug interaction | F_(2,22)_=2.238 | 0.1304 |
|  | Within subject: AD | F_(1.597,17.56)_=5.452 | 0.0193***** |
|  | Between subject: Drug | F_(1,11)_=0.0602 | 0.8107 |
| S1H: OF center entries | Unpaired t-test | t_(11)_=0.3094 | 0.7628 |
| S1I: SoPT habituation | Unpaired t-test | t_(11)_=1.229 | 0.2447 |
| S1J: SoPT entries | Two Way Mixed ANOVA |  |  |
|  | Zone x Drug interaction | F_(1,11)_=0.6806 | 0.4269 |
|  | Within subject: Zone | F_(1,11)_=1.071 | 0.3230 |
|  | Between subject: Drug | F_(1,11)_=0.0462 | 0.8337 |
| S1K: SuPT consumption | Two Way Mixed ANOVA |  |  |
|  | Liquid x Drug interaction | F_(1,11)_=1.051 | 0.3273 |
|  | Within subject: Liquid | F_(1,11)_=0.1386 | 0.7168 |
|  | Between subject: Drug | F_(1,11)_=0.0279 | 0.8705 |
| S1L: EPM entries | Unpaired t-test | t_(11)_=1.424 | 0.1823 |
| **Figure S2:** Early abstinence and late abstinence negative affect | | | |
| S2A: Inact pokes seeking | Two Way Mixed ANOVA |  |  |
|  | AD x Drug interaction | F_(1,14)_=0.0092 | 0.9248 |
|  | Within subject: AD | F_(1,14)_=17.50 | 0.0009***** |
|  | Between subject: Drug | F_(1,14)_=8.060 | 0.0131***** |
| S2B: OF distance | Two Way Mixed ANOVA | | |
|  | AD x Drug interaction | F_(1,14)_=0.5402 | 0.4745 |
|  | Within subject: AD | F_(1,14)_=0.0242 | 0.8786 |
|  | Between subject: Drug | F_(1,14)_=0.1561 | 0.6987 |
| S2C: SoPT habituation (%) | Two Way Mixed ANOVA |  |  |
|  | AD x Drug interaction | F_(1,14)_=0.5149 | 0.4848 |
|  | Within subject: AD | F_(1,14)_=0.4074 | 0.5336 |
|  | Between subject: Drug | F_(1,14)_=0.0172 | 0.8975 |
| S2D: EPM entries | Two Way Mixed ANOVA |  |  |
|  | AD x Drug interaction | F_(1,14)_=2.575 | 0.1309 |
|  | Within subject: AD | F_(1,14)_=2.209 | 0.1594 |
|  | Between subject: Drug | F_(1,14)_=0.0288 | 0.8677 |
| **Figure S3:** Housing condition and negative affect | | | |
| S3A: OF distance | Unpaired t-test (Single, Pair) | t_(14)_=0.2889 | 0.7769 |
| S3B: OF center entries | Mann Whitney test (Single, Pair) | U=22 | 0.3148 |
| S3C: SoPT habituation (%) | Unpaired t-test (Single, Pair) | t_(14)_=0.3993 | 0.6957 |
| S3D: SoPT entries | Unpaired t-test (Single, Pair) | t_(14)_=0.5789 | 0.5719 |
| S3E: SuPT consumption | Unpaired t-test (Single, Pair) | t_(14)_=1.639 | 0.1235 |
| S3F: EPM entries | Unpaired t-test (Single, Pair) | t_(14)_=1.070 | 0.3026 |

Table outlines statistical test used, results, and p-value for data included in Figures S1, S2, and S3. *p<0.05, #p<0.1. Abbreviations: abstinence day (AD), average (avg), elevated plus maze (EPM), open field (OF), oxycodone (Oxy), saline (Sal), self-administration (SA), social preference test (SoPT), somatic withdrawal (SWD), sucrose preference test (SuPT).

**Table S2. Correlation analysis of oxycodone (Oxy) consumption and spontaneous somatic withdrawal signs on abstinence day 1**

| **Variable 1** | **Variable 2** | **n** | **Pearson’s r** | **p** |
| --- | --- | --- | --- | --- |
| Oxy consumed (mg/kg) | Weight change | 7 | 0.0425 | 0.928 |
| Oxy consumed (mg/kg) | Somatic withdrawal score | 7 | 0.5462 | 0.205 |
| Oxy consumed (mg/kg) | Grooming bouts | 7 | 0.1721 | 0.712 |
| Oxy consumed (mg/kg) | Wet dog shakes | 7 | 0.7054 | 0.077# |
| Oxy consumed (mg/kg) | Teeth chatters | 7 | 0.4680 | 0.290 |
| Oxy consumed (mg/kg) | Jumps | 7 | -0.1357 | 0.772 |
| Oxy consumed (mg/kg) | Digging bouts | 7 | 0.5931 | 0.160 |

Table outlines the correlation between total oxycodone consumed (mg/kg) over 10 d of self-administration and weight change (pre-self-administration versus abstinence day (AD) 1), total somatic withdrawal score on AD1, and the number of grooming bouts, wet dog shakes, teeth chatters, jumps, and digging bouts on AD1. Data are included in Figures 1 and S1. #p<0.08. Abbreviations: abstinence day (AD), oxycodone (Oxy).

**Table S3. Comparison of male and female rats during oxycodone self-administration and seeking tests**

| **Figure** | **Statistical Test** | **F** | **p-value** |
| --- | --- | --- | --- |
| SA: Infusions | Two Way Mixed ANOVA |  |  |
|  | Day x Sex interaction | F_(2.533,38.00)_=1.539 | 0.2243 |
|  | Within subject: Day | F_(2.533,38.00)_=5.870 | 0.0034* |
|  | Between subject: Sex | F_(1,15)_=0.4457 | 0.5145 |
| SA: Active nose pokes | Two Way Mixed ANOVA | | |
|  | Day x Sex interaction | F_(2.494,37.40)_=1.336 | 0.2771 |
|  | Within subject: Day | F_(2.494,37.40)_=2.306 | 0.1026 |
|  | Between subject: Sex | F_(1,15)_=0.6386 | 0.4367 |
| SA: Inactive nose pokes | Two Way Mixed ANOVA | | |
|  | Day x Sex interaction | F_(2.234, 33.51)_=0.7880 | 0.4757 |
|  | Within subject: Day | F_(2.234, 33.51)_=1.096 | 0.3515 |
|  | Between subject: Sex | F_(1, 15)_=0.2427 | 0.6294 |
| Seeking: Incubation Score | Unpaired t-test (male, female) | t_(7.441)_=0.0355 | 0.9726 |

Table outlines additional statistical analyses on rats that self-administered oxycodone for 6 h/d for 10 d. This self-administration (SA) analysis combined animals from Figures 1 and 2 (9 females and 8 males in total). No significant main effect of sex was detected for number of infusions, active nose pokes, or inactive nose pokes. The seeking analysis was run on animals that self-administered oxycodone and underwent seeking tests on both AD1 and AD40 (Figure 2). There was no significant difference in the incubation score in male (n=4) and female (n=6) rats. *p<0.05. Abbreviations: self-administration (SA).

**Table S4. Rat equivalent daily dosing of oxycodone (Oxy) relative to human dosing for pain management**

| **Human Oxy Dosing** | **Daily Human Oxy Intake** | **K_m_** (for rats) | **Rat Oxy Equivalent Dose** | **Rat Oxy Dosing** | **Average Oxy Intake during SA** |
| --- | --- | --- | --- | --- | --- |
| 20-60 mg per day | 0.33-1 mg/kg | 6.2 | 2.05-6.2 mg/kg | 0.41-2.79 mg per day | 1.71 mg per day |

Table S4 summarizes the conversion of clinically relevant human oxycodone (Oxy) dosing to rat-equivalent doses using allometric scaling (1). Human dosing was based on a typical pain management regimen of 5–15 mg administered every 6 hours (20–60 mg/day), corresponding to 0.33–1 mg/kg/day assuming a 60 kg individual. Rat-equivalent doses were calculated by multiplying the human mg/kg dose by the rat K_m_ factor (6.2), yielding 2.05–6.2 mg/kg/day. This was converted to a total daily dose using an average rat body weight of 0.2-0.45 kg (0.41–2.79 mg/day). Average oxycodone intake during self-administration was calculated from infusion data in this study (mean=52.6 infusions/day) and a mean body weight of 0.325 kg, resulting in an average intake of 1.71 mg/day. Notably, this level of intake falls within the range of rat-equivalent doses derived from clinically relevant human pain management regimens.

**Supplemental Material Citation (Table S4)**

1. Jacob S, Nair AB, Morsy MA. Dose conversion between animals and humans: A practical solution. Indian Journal of Pharmaceutical Education and Research. 2022; 56(3):600-607.
